# Induction of the Staphylococcal Proteolytic Cascade by Antimicrobial Fatty Acids in Community Acquired Methicillin Resistant *Staphylococcus aureus*

**DOI:** 10.1371/journal.pone.0045952

**Published:** 2012-09-24

**Authors:** Benjamin Arsic, Yue Zhu, David E. Heinrichs, Martin J. McGavin

**Affiliations:** 1 Department of Microbiology and Immunology, Western University, London, Ontario, Canada; 2 Centre for Human Immunology, Western University, London, Ontario, Canada; National Institutes of Health, United States of America

## Abstract

Community acquired methicillin resistant *Staphylococcus aureus* (CA-MRSA), and the USA300 strain of CA-MRSA in particular, are known for their rapid community transmission, and propensity to cause aggressive skin and soft tissue infections. To assess factors that contribute to these hallmark traits of CA-MRSA, we evaluated how growth of USA300 and production of secreted virulence factors was influenced on exposure to physiologic levels of unsaturated free fatty acids that would be encountered on the skin or anterior nares, which represent the first sites of contact with healthy human hosts. There was a sharp threshold between sub-inhibitory and inhibitory concentrations, such that 100 µM sapienic acid (C16∶1) and linoleic acid (C18∶1) were sufficient to prevent growth after 24 h incubation, while 25 µM allowed unrestricted growth, and 50 µM caused an approximate 10–12 h lag, followed by unimpeded exponential growth. Conversely, saturated palmitic or stearic acids did not affect growth at 100 µM. Although growth was not affected by 25 µM sapienic or linoleic acid, these and other unsaturated C16 and C18 fatty acids, but not their saturated counterparts, promoted robust production of secreted proteases comprising the Staphylococcal proteolytic cascade. This trait was also manifested to varying degrees in other CA-MRSA, and in genetically diverse methicillin susceptible *S. aureus* strains. Therefore, induction of the Staphylococcal proteolytic cascade by unsaturated fatty acids is another feature that should now be evaluated as a potential contributing factor in the aggressive nature of skin and soft tissue infections caused by USA300, and as a general virulence mechanism of *S. aureus*.

## Introduction

*Staphylococcus aureus* has a dichotomous relationship with humans. It is a ubiquitous commensal that persistently colonizes 25%–30% of the human population [1], yet it also has a broad arsenal of virulence factors [2], which enable it to be a leading cause of infections, ranging from relatively mild skin and soft tissue infections, to severe and life-threatening conditions such as necrotizing pneumonia, osteomyelitis, and infective endocarditis [2], [3]. The preferred site of colonization is the anterior nares, and infections are typically a consequence of autologous nasal carriage [4]. Significant effort has therefore been directed towards identifying host and microbial factors that determine the carriage or non-carriage status [1], [5], [6], [7], [8], [9], [10], [11], [12], and in this context, our study is based on two broad assumptions. First, in order to maintain a persistent carrier status, *S. aureus* must endure innate defense mechanisms of the skin and mucosal surfaces, and from this, it follows that highly invasive pandemic strains of *S. aureus* should possess effective means of responding to these innate defense mechanisms.

The USA300 strain of community acquired methicillin resistant *S. aureus* (CA-MRSA) is well suited for testing these tenets of virulence and transmission. For approximately 30 years after the emergence of methicillin resistance, MRSA were restricted to the hospital environment, and these hospital-associated MRSA evolved by acquiring resistance to multiple antimicrobial agents [13], [14], [15], [16]. However, beginning in the 1990’s, the epidemiology of MRSA colonization and infection has undergone a paradigm shift with the rapid emergence and pandemic community transmission of the USA300 strain of CA-MRSA, which is known for causing aggressive skin and soft tissue infections that can progress to fatal complications if not rapidly treated [17], [18]. USA300 is now the leading cause of visits to hospital emergency departments in North America, for treatment of skin infections [19], and is displacing less virulent HA-MRSA, potentially aided by its ability to establish asymptomatic nasal carriage in health care workers [20]. USA300 is more easily transmitted to household contacts compared to other *S. aureus* genetic backgrounds [21], and this study which surveyed the inguinal area in addition to the anterior nares, would have missed 51% of MRSA colonized persons if it had been conducted on a nares-only basis [21], which supports the contention that the rampant community transmission of USA300 could be due in part to a superior ability to persist on skin surfaces. A portion of this success is attributed to the arginine catabolism mobile element ACME, which uniquely confers resistance of USA300 to antimicrobial polyamines [22], and has been proposed to facilitate persistence on skin through catabolism of arginine with concomitant release of ammonia to neutralize acidic pH [23], [24], [25].

Another barrier to persistence of bacteria on skin is the antimicrobial properties of sebum [26], [27], which is a liquid phase lipid mixture secreted from the sebaceous glands, consisting of approximately 28% free fatty acids, 32% triglycerides, 25% wax esters, and 11% squalene [28]. In sebum triglycerides and free fatty acids, the major component is sapienic acid (C16∶1Δ6), which is an isomer of palmitoleic acid (C16∶1Δ9), and exhibits the primary antimicrobial activity [27]. The importance of sapienic acid as an innate defense mechanism is evident in atopic dermatitis, where the skin is deficient in this fatty acid [29], and there is a near 100% recovery of *S. aureus* from the skin of atopic dermatitis patients. *S. aureus* is also exposed to antimicrobial fatty acids in colonization of the anterior nares, where palmitoleic (sapienic) and linoleic acid (C18∶2) were identified as the major unsaturated free fatty acids (uFFA) in human nasal secretions [30]. Linoleic acid also accumulates to high levels within *S. aureus* abscesses [31], and abscess formation is a hallmark of *S. aureus* infection of the skin and soft tissues.

Although exposure to antimicrobial fatty acids would be one of the first signals encountered by *S. aureus* during colonization of the skin or anterior nares, studies that have assessed the response of CA-MRSA to host-specific environmental signals have focused on stresses related to growth in blood, or phagocytosis by neutrophils [32], [33]. Therefore, the goal of this study was to evaluate how growth and production of secreted virulence factors by USA300 is influenced by exposure to physiologic levels of uFFA. Herein, we describe the robust induction of the Staphylococcal proteolytic cascade pathway (SPC) in response to sub-inhibitory concentrations of uFFA in USA300 and other strains of CA-MRSA, and this response was also evident to varying degrees in clinical MSSA. The Staphylococcal proteolytic cascade is comprised of a metalloprotease Aureolysin, which is needed to activate the SspA serine protease, which in turn activates the SspB cysteine protease that is co-expressed with SspA in the *sspABC* operon [34], [35], [36], [37], [38]. We discuss the implications of this environmental signal-response pathway, and its potential impact on colonization, transmission, and the aggressive nature of skin and soft tissue infections caused by CA-MRSA.

## Materials and Methods

### Strains and Growth Conditions

Bacterial strains and plasmids used in this study are defined in Table 1. Cultures were maintained as frozen stocks (–80°C) in 20% glycerol, and streaked on TSB agar when required. TSB was supplemented, when necessary, with 10 µg/mL erythromycin or 2 µg/mL tetracycline for propagation of strains bearing resistance markers.

**Table 1:** *S. aureus* strains and plasmids used in this study.

Strain	Description	Source/Reference	
RN4220	Restriction deficient lab strain	[42]	
DU5969	RN4220*aur::lacZ*	[48]	
USA300 LAC	Hypervirulent pandemic CA-MRSA Los Angeles county clone, clonal complex CC8 *spa* t008	[77], [78]Barry Kreiswirth	
USA300	USA300 LAC cured of antibiotic resistance plasmid	This study	
USA300*aur::lacZ*	*aur::lacZ* from DU5969 transduced into USA300	This study	
USA300*sspABC*	Replacement of *sspABC* in USA300 with Tc^r^ cassette, using pMJ232	This study	
USA300*aur*	Transduction of *aur::lacZ* into USA300	This study	
USA300*sspABCaur*	Transduction of *aur::lacZ* into USA300*sspABC*	This study	
USA400	CA-MRSA fatal pediatric bacteremia, CC1 *spa* t127	[79]	
MSSA476	CA-MSSA closely related to USA400; pediatric osteomyelitis	[75]	
Newman	MSSA clinical isolate; routinely used in virulence studies, CC8 *spa* t008	[80], [81]	
WBG10049	Southwest Pacific Clone of CA-MRSA, CC30 *spa* t019	[82]	
MRSA252	HA-MRSA; CC30, *spa* t016	[83]	
UAMS-1	MSSA osteomyelitis, CC30 *spa* t033	[84]	
SRI-138	MSSA dermatitis CC45 *spa* t065	[85]	
SRI-109	MSSA dermatitis; CC45 *spa* t015	[85]	
PED1–75	MSSA pediatric dermatitis; CC5 *spa* t002	[85]	
SRI-116	MSSA dermatitis; CC1 *spa* t7404	[85]	
SRI-142	MSSA dermatitis; CC1 *spa* t4938	[85]	
PED2–1	CA-MRSA pediatric dermatitis; CC97 *spa* t7398	[85]	
PED1-53	MSSA pediatric dermatitis; CC8 *spa* t008	[85]	
L528	MSSA infective endocarditis; CC30 *spa* t033	[52]	
PED1-37	MSSA pediatric dermatitis; CC398 *spa* t937	[85]	
pMAD	Shuttle vector for construction of mutations in Gram-positive bacteria	[41]	
pDG1514	Source of Tc^r^ cassette	[86]	
pMJ232	pMAD containing Tc^r^ cassette from pDG1514, flanked by *Bam*HI-[*sspA-*5P]-*Mlu*I and *EcoR*I-[*sspC-*3P]-*Bgl*II	This study	

### Generation of Plasmid-cured USA300

USA300 LAC was a generous gift from Dr. B. Kreiswirth. To facilitate mutagenesis in the USA300 genetic background, USA300 LAC was cured of the 27-kb plasmid [39], yielding USA300, which is sensitive to erythromycin, kanamycin and neomycin, using the method previously described [40]. The plasmid cured USA300 LAC is referred to as USA300 throughout.

### Construction of USA300Δ*sspABC*

The *sspABC* operon encodes the SspA serine protease and Staphopain B cysteine protease SspB. For construction of USA300Δ*sspABC::tc*, three DNA segments were assembled in pMAD, consisting of a 702-nt *sspA* 5′-flanking segment *Bam*HI-[*sspA-*5P]-*Mlu*I, a 2.1 kb *Pst*I-*Eco*RI restriction fragment containing the Tc resistance cassette from pDG1514, and a 760-nt *sspC* 3′-flanking segment *Eco*RI-[*sspC-*3P]-*Bgl*II. The 702-nt *sspA*-5P flanking sequence was amplified by PCR with primers *sspA-*5PF (5′-cgc***ggatcc***CGGTAAAGGATTTGTAAGGATTTCC-3′) and *sspA-*5PR (5′-gcg***acgcgt***TTGCTGCTGGAGAACTCACAAGTG-3′), where the lower case residues in bold italics represent added *Bam*HI and *Mlu*I restriction sites. Similarly, the 760 nt *sspC-*3P flanking segment was amplified with primers *sspC-*3PF (5′-ccc***gaattc***CAATTTCTCACCAGCTCG-3′) and *sspC-*3PR (5′-gga***agatct***GTAGGTGAAGACCAAATCCCTCG-3′), incorporating respective *Eco*RI and *Bgl*II sites. After plasmid assembly in *E. coli* DH5α, the resulting plasmid pMJ232 was transferred into *S. aureus* Newman via electroporation, using *S. aureus* RN4220 as an intermediate host. Construction of the Δ*sspABC::tc* deletion mutation was conducted following protocols established for use with pMAD [41]. The Δ*sspABC::tc* deletion was confirmed by PCR with two primer pairs; one which flanks the external boundaries of the 2.6-kb deletion, and the second which anneals within the deleted segment, and yields a product only with wild type genomic DNA. The Δ*sspABC::tc* mutation was then transferred from strain Newman to USA300 using a phage φ85 transducing lysate.

### Molecular Biology Protocols

Protocols for plasmid construction in *E. coli* DH5α and genetic manipulation of *S. aureus*, including isolation of plasmid and genomic DNA, electroporation, and phage transduction have been described previously [35], [36], [42]. Restriction enzymes and DNA ligase were purchased from New England BioLabs, and AmpliTaq Gold DNA polymerase was purchased from Life Technologies. DNA amplification was conducted using a PTC-100 Thermal Controller (MJ Research). The integrity of cloned PCR products was confirmed by sequencing of plasmid constructs at the London Regional Genomics facility of the Robarts Research Institute.

### TCA Precipitation of Proteins, SDS-PAGE, Western Blotting and Mass Spectrometry

For SDS-PAGE analyses, proteins in the cell-free culture supernatant were precipitated by mixing with an equal volume of ice-cold 20% TCA, washed in ice cold 70% ethanol, then air dried and dissolved in SDS-PAGE reducing buffer as described previously [37]. The culture density (OD_600_) was determined prior to preparation of cell-free culture supernatant, and for analysis of secreted protein profiles, TCA precipitated protein derived from 2.0 OD_600_ units of culture was applied to each lane of a 12% acrylamide gel. Identification of Coomassie-Blue stained proteins was conducted at the London Regional Proteomics Centre at Western University. Protein bands were excised using an Ettan™ Spot Picker, and processed for mass spectrometry using a Waters MASSPrep Automated Digester as described [43]. Processed samples were spotted on MALDI plates and analyzed on an Applied Biosystems 4700 Proteomics Analyzer. Data were acquired and processed using 4000 Series Explorer and Data Explorer (Applied Biosystems), and the peptide fingerprints were compared to the NCBInr database for Gram-positive bacteria, using the MASCOT search engine.

For Western blotting, a volume of cell-free culture supernatant corresponding to 0.02 to 0.05 OD_600_ units was mixed directly with SDS-PAGE reducing buffer, and applied to 12% polyacrylamide gels. Rabbit polyclonal antiserum specific for SspA and Aur proteases was used as described previously [36], [37], and rabbit polyclonal antiserum specific for Hla was purchased from Sigma. Blots were developed with donkey anti-rabbit IgG IR800 conjugate (Rockland Immunochemicals Inc.), and images were captured using an Odyssey infrared imager from LiCor Biosciences.

### Influence of Fatty Acids on Growth of *S. aureus*

Sapienic acid (cis-6-Hexadecenoic acid; 16∶1ω10) was purchased from Matreya LLC. Palmitic acid (hexadecanoic acid; 16∶0), palmitoleic acid (*cis-9*-hexadecenoic acid; 16∶1), stearic acid (octadecanoic acid; 18∶0), oleic acid (*cis-9*-octadecenoic acid; 18∶1), linoleic acid (*cis, cis-9,12*-octadecadienoic acid; 18∶2), linolenic acid (*cis,cis,cis-9,12,15*-octadecatrienoic acid; 18∶3) and glycerol monolaurate were all purchased from Sigma. Prior to supplementing TSB media, fatty acids were first mixed with an equal volume of DMSO, and then diluted in TSB to a working stock concentration of 5 mM.

For growth analyses, bacteria from single colonies on TSB agar were inoculated into culture tubes containing 3 mL of antibiotic free TSB, and grown overnight at 37°C on an orbital shaker, followed by measurement of OD_600_. A 25 mL volume of TSB, supplemented with fatty acid where indicated, was then inoculated to achieve a starting OD_600_ of 0.01, and the cultures were grown 37°C on an orbital shaker incubator, set at 180 rpm. Measurements of OD_600_ were taken at hourly intervals, and at set time points, samples were withdrawn for recovery of cell-free culture supernatant. All growth analyses were conducted in triplicate, from three separate cultures.

### Protease and β-galactosidase Assays

Total protease activity in cell free culture supernatant was assayed with FITC-casein substrate (Sigma Type II). Prior to assay, the supernatant samples were normalized by dilution with sterile water as needed, to adjust for minor differences in cell density of the stationary phase cultures at time of harvest. Triplicate aliquots of the normalized culture supernatant (490 µL) were mixed with 460 µL of incubation buffer (40 mM Tris-HCl pH 7.4, 300 mM NaCl, 20 mM CaCl_2_, and 2 mM L-cysteine) and 50 µL of 0.2% w/v FITC-casein. Blanks were prepared using 490 µL of sterile culture supernatant. The samples were incubated at 37°C in the dark for 2 h. Trichloroacetic acid was then added to 4% w/v to stop the reaction, and the samples were centrifuged at maximum speed for 15 m to pellet undigested casein. The supernatant was then mixed with an equal volume of 0.5 M Tris-HCl, pH 8.5, and after transfer to Optilux black clear bottom microtitre plates (BD Falcon), fluorescence was quantified on a Cary Eclipse Fluorometer using excitation at 485 nm and emission at 535 nm.

For β-galactosidase reporter assays, USA300*aur::lacZ* was cultured in TSB or TSB supplemented with palmitic or palmitoleic acid, and 1 mL aliquots were withdrawn at 5, 6, 7 and 8 h of growth. After washing in 1 mL of ice cold PBS, the cells were resuspended in 1 mL of PBS, and transferred to Lysing Matrix B tubes containing 0.5 ml of 0.1 mm silica beads (MP Biomedicals). The tubes were placed in a FastPrep120 homogenizer (Savant Biosystems) for 20 s at a setting of 6.5. After centrifugation, a 20 µl aliquot of each cell-lysate was added to triplicate wells of Optilux microtitre plates containing 50 µM fluorescein-di-β-D-galactopyranoside (AnaSpec) in 150 µL of 0.1 M sodium phosphate buffer pH 7.3, 1 mM MgCl_2_, and 45 mM β-mercaptoethanol. After incubation in the dark for 30 min, the assay was terminated by adding 50 µL of 0.2 M Na_2_CO_3_ to each well, and fluorescence was read using the Varian Cary Eclipse Fluorescence Spectrophotometer (Excitation/Emission = 490 nm/520 nm).

## Results

### Influence of Fatty Acids on Growth of *S. aureus* USA300

A recent study reported that the median concentrations of palmitoleic acid (C16∶1) and linoleic acid (C18∶2) were 48 µM and 16 µM respectively in human nasal secretions [30]. Moreover, Kenny *et al.* reported that 10 µM linoleic acid (LA) was not growth inhibitory, whereas 100 µM LA inhibited growth of *S. aureus* strain MRSA 252 [44]. We therefore chose this range of concentrations to assess the influence of LA and other saturated and unsaturated fatty acids on growth of USA300, a clinically important strain notorious for its ability to cause serious skin and soft tissue infections.

**Figure 1:**
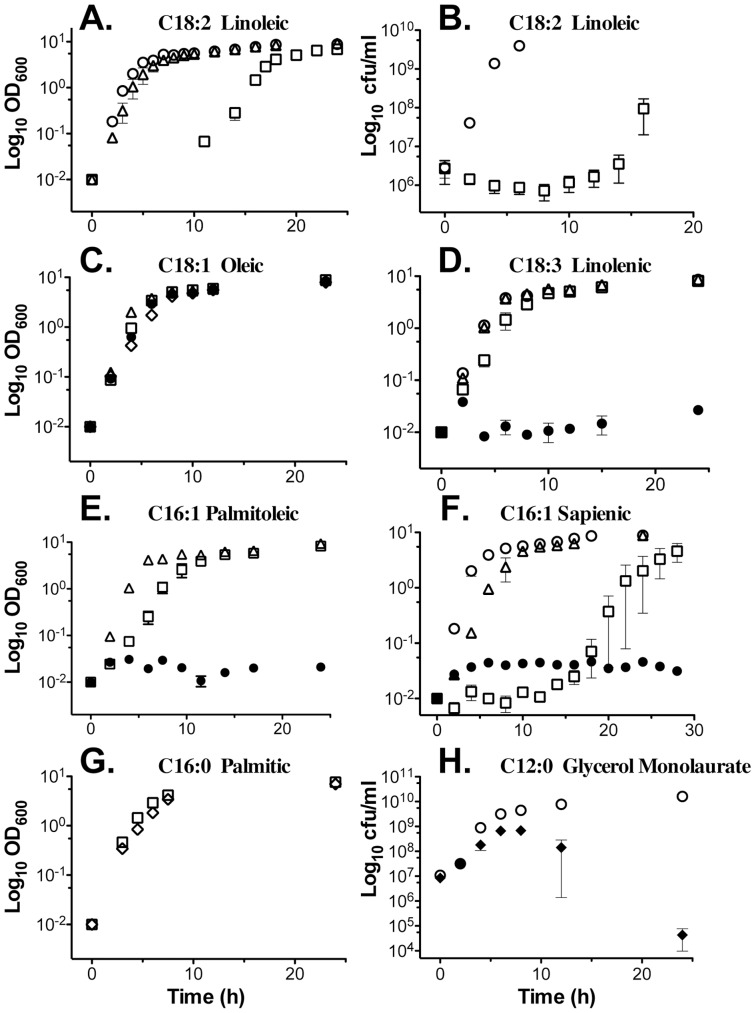
Influence of fatty acids on growth of USA300. Each point represents the mean of OD_600_ (A, C, D-G) or cfu/ml determination (B, H) from triplicate flasks of USA300 grown in TSB supplemented with the indicated amount of fatty acid; (○), TSB only; (▵), 25 µM; (□), 50 µM; (•), 100 µM; (⋄), 200 µM; (♦), 250 µM. Lauric acid (C12∶0) was provided in the form of triacylglycerol-monolaurate. Y-axes, OD_600_ or cfu/ml; X-axis, growth time (h).

Growth of USA300 was not influenced by up to 200 µM saturated stearic acid (C18∶0; data not shown), or by 25 µM linoleic acid (Fig. 1A). However there was a sharp boundary between sub-inhibitory and growth inhibitory concentrations of linoleic acid (LA), and a concentration of 50 µM consistently promoted a 12 h lag phase, followed by unimpeded exponential growth. When viable cell counts were measured, there was a slight loss of viability over the first 8 h of incubation in TSB containing 50 µM LA, after which the culture began to recover, with initiation of exponential growth at 12 h (Fig. 1B). As reported by Kenny *et al.* [44], there was no growth at 100 µM LA (data not shown), and this was also noted with linolenic, palmitoleic, and sapienic acid (Fig. 1D, E and F). The growth inhibition of sapienic acid closely resembled linoleic acid, with a 50 µM concentration promoting an extended lag phase followed by unimpeded exponential growth (Fig. 1F). The only exception to growth inhibition by uFFA was oleic acid, which was not inhibitory up to 200 µM, and this is consistent with data from Parsons *et al.*, where *S. aureus* was cultured with 500 µM oleic acid, albeit with use of BSA as a carrier [45].

Saturated C16∶0 palmitic acid was not inhibitory up to 200 µM (Fig. 1G), confirming that antimicrobial activity is restricted to uFFA. A possible exception is lauric acid C12∶0, which has been described as an antimicrobial component of human sebum [46]. We therefore tested glycerol monolaurate as a source of C12∶0, and found that growth was not affected by up to 100 µM (data not shown), whereas 250 µM permitted growth into late exponential phase, after which there was a rapid decline in cell viability (Fig. 1H). We conclude that unsaturated sapienic, palmitoleic and linoleic acid are the most effective inhibitors of *S. aureus* growth, and the threshold between sub-inhibitory and growth inhibitory concentration occurs between 25 µM and 50 µM, which approximates the median concentrations of linoleic and palmitoleic acid in human nasal secretions.

### Expression of Secreted Proteins is Altered by uFFA

**Figure 2:**
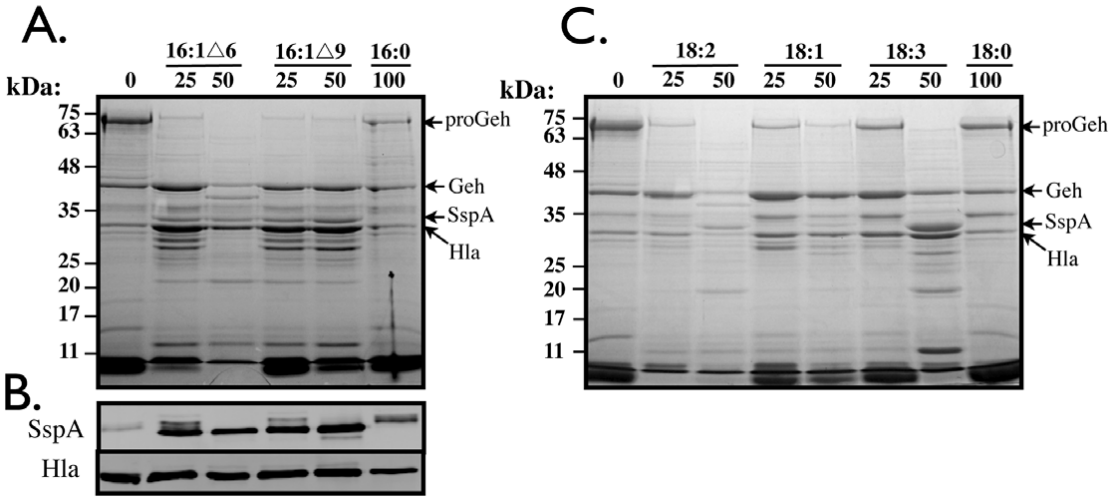
SDS-PAGE of secreted proteins (A, C) and Western blot for detection of SspA and Hla (B), in culture supernatant of USA300 after growth for 18–24 h in the presence of C16 (A) or C18 (C) fatty acids. Cultures were grown with the indicated amounts of C16∶1▵6 (sapienic acid), C16∶1▵9 (palmitoleic acid), C16∶0 (palmitic acid), C18∶2 (linoleic acid), C18∶1 (oleic acid), C18∶3 (linolenic acid) or C18∶0 (stearic acid) fatty acids. Proteins in the cell-free culture supernatant were precipitated in ice-cold TCA, and after solubilization in SDS-PAGE reducing buffer, protein equivalent to 2.0 OD_600_ units of culture supernatant was loaded in each lane (A and C). For Western blot (C), 0.02 OD_600_ units of cell free culture supernatant were subjected directly to SDS-PAGE, prior to detection with specific antisera (see Materials and Methods).

As a means to understand the response of *S. aureus* to the various fatty acids, we examined protein expression profiles. While no significant changes were readily detected in whole cell lysate (data not shown), we readily observed that the profile of secreted proteins produced by USA300 was profoundly altered after growth to stationary phase in the presence of unsaturated sapienic (C16∶1Δ6), palmitoleic (C16∶1Δ9), linoleic (C18∶2), oleic (18∶1), and linolenic (C18∶3) fatty acids (Fig. 2), whereas 100 µM saturated palmitic acid (C16∶0) or stearic acid (C18∶0) had no influence on growth or profile of secreted proteins compared to TSB alone (Fig. 2A and C). These changes were clearly evident even at 25 µM concentrations of uFFA that did not alter growth kinetics. As determined by mass spectrometry analyses of selected proteins, the most significant change upon exposure to uFFA was the appearance of a new protein corresponding to the SspA serine protease, which is co-expressed with the SspB cysteine protease in the staphylococcal serine protease operon *sspABC* [38], and this was especially evident with the C16∶1 fatty acids, and 50 µM linolenic acid. This was typically accompanied by loss or diminished production of a 72-kDa precursor isoform of glycerol ester hydrolase, together with accumulation of 40 kDa mature Geh.

A Western blot confirmed that production of SspA was up-regulated by the C16∶1 fatty acids, whereas saturated C16∶0 had no affect, with SspA remaining in its slightly larger precursor isoform (Fig. 2B). Conversely, production of Hla was not influenced by either saturated or unsaturated fatty acids. From these data, we conclude that uFFA primarily influence the production of secreted proteases, concomitant with maturation of proGeh.

### The Staphylococcal Proteolytic Cascade (SPC) is Induced by uFFA

**Figure 3:**
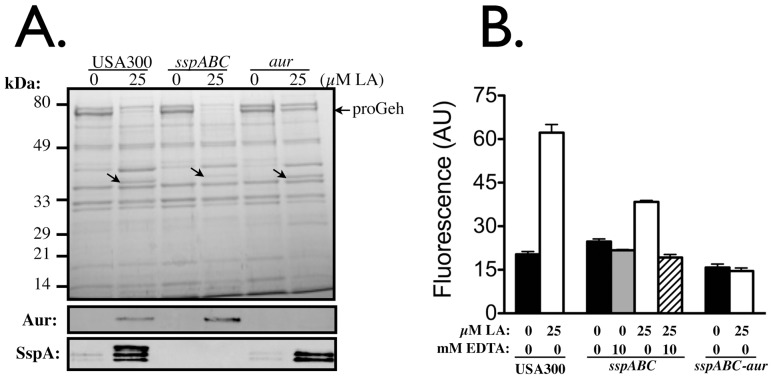
SDS-PAGE and Western blot analyses of secreted proteins produced by USA300 and isogenic variants after 8 h of growth in TSB, or TSB supplemented with 25 µM linoleic acid (A), and assay of total protease activity in culture supernatant (B). For (A), protein loading was 2.0 OD_600_ units for Coomassie staining, and 0.02 OD_600_ units for Western blots, which were developed with primary antibody specific for Aur, and SspA as indicated. Arrows on the Coomassie stained gel indicate the selective induction of secreted proteases in response to linoleic acid. The arrow on the right margin indicates the position of proGeh. In (B), total protease activity in 8 h culture supernatant of USA300 and isogenic variants was determined with FITC-casein substrate. Cultures were grown with 25 µM linoleic acid as indicated, and assay buffer was supplemented with 10 mM EDTA where indicated, to inhibit metalloprotease. Data are reported as fluorescence emission at 535 nm (ε_535_), measured in arbitrary fluorescence units.

The SPC is initiated by autocatalytic activation of the metalloprotease Aureolysin [36]. However, we have observed that mature Aureolysin is unstable, and its secretion and maturation precedes the appearance of proSspA and proSspB in culture supernatant [36]. We therefore cultured USA300 and isogenic *aur* or *sspABC* derivatives in TSB for 8 h, to assess the influence of linoleic acid at an earlier time point (Fig. 3). In terms of the impact on secreted proteins, our data clearly establish that the primary effect of linoleic acid is to induce protease expression, as evident from the appearance of a new protein corresponding to Aur in USA300*sspABC*, and induction of SspA in USA300*aur*, while both proteins are produced in wild type USA300 (Fig. 3A). The only other obvious changes were alterations in the relative amounts of 72-kDa proGeh in USA300 and USA300*sspABC* after growth with linoleic acid, but this was less evident in USA300*aur*. This is consistent with a role for Aur in processing of proGeh, as reported for orthologous metalloprotease and lipase proteins in *S. hyicus* [47].

Enhanced production of secreted protease was confirmed by assay of total protease activity in culture supernatant (Fig. 3B), which was significantly increased when USA300 was cultured with 25 µM LA. Protease activity was also enhanced, although to a lesser extent, when USA300*sspABC* was cultured with 25 µM LA, and this activity was inhibited with EDTA, confirming that metalloprotease is induced by LA. No increase in activity was evident when USA300*sspABC-aur* was cultured with LA, even though *scpA* encoding the Staphopain A cysteine protease was not targeted for disruption. Therefore, it appears that the influence of uFFA is specific to *aur* and the *sspABC* genes that comprise the Staphylococcal Proteolytic Cascade (SPC), but does not affect Staphopain A (ScpA), which does not comprise part of the staphylococcal proteolytic cascade, because it undergoes autocatalytic activation independently of other protease functions [35].

**Figure 4:**
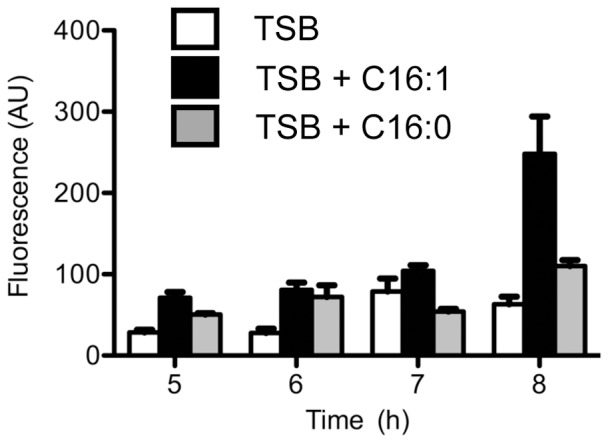
β-galactosidase reporter gene assay in cell lysate of USA300*aur* after growth for 5–8 h in TSB, or TSB supplemented with 25 µM palmitic (C16∶0) or palmitoleic (C16∶1) acid.

To confirm that these changes occur at the transcriptional level, we took advantage of the *aur::lacZ* fusion created by inactivation of *aur*, which places *lacZ* under transcriptional control of the *aur* promoter [48]. We cultured USA300*aur* in either TSB, or TSB supplemented with 25 µM palmitic (C16∶0) or palmitoleic acid (C16∶1), taking samples between 5 and 8 h, which as shown in Fig. 1E and 1G, corresponds to the transition between exponential growth and post-exponential phase. Assay of β-galactosidase activity in the total cell lysates revealed a clear induction after 8 h when USA300*aur* was cultured with 25 µM palmitoleic acid (Fig. 4). This experiment was repeated with replicates of three cell lysates for each growth condition, and after 8 h of growth, palmitoleic acid promoted significantly greater β-galactosidase activity compared to palmitic acid (p = 0.037) or TSB alone (p = 0.004), and there was no significant difference comparing growth in TSB alone versus TSB supplemented with palmitic acid (p = 0.090). Cumulatively, these data establish that uFFA induce expression of secreted proteases comprising the Staphylococcal Proteolytic Cascade.

### Induction of the Staphylococcal Proteolytic Cascade by uFFA is a Common but Variable Trait of *S. aureus* Clinical Isolates

**Figure 5:**
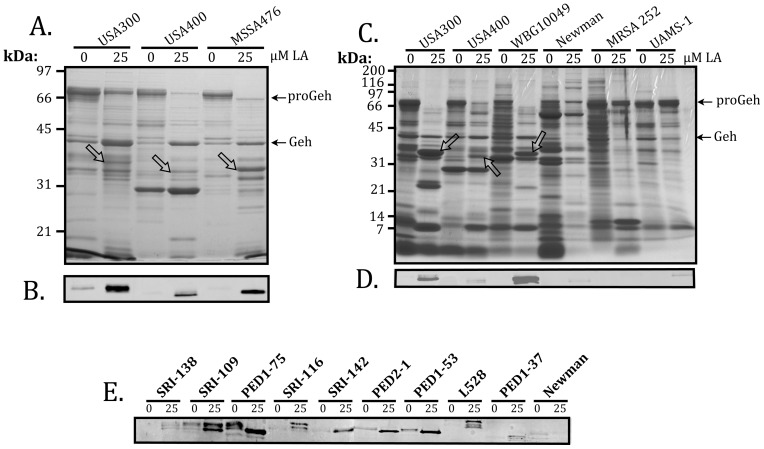
SDS-PAGE and Coomassie staining (A and C), or Western blot for detection of SspA (B, D and E), in cultures of *S. aureus* grown in TSB containing 0 or 25 µM linoleic acid (LA) as indicated. Protein loading was 2.0 OD_600_ units for SDS-PAGE, and 0.02 OD_600_ units for Western blot. The *S. aureus* strains are defined in Table 1. Arrows and labels on the right margins of panels A and C indicate the location of 72 kDa glycerol ester hydrolase precursor (proGeh) and mature lipase (Geh), while arrows on the protein gels point to SspA protein that is induced in response to 25 µM LA. SspA exhibits some expected variation in size, being comprised of 327 amino acids in USA400 (MW_0932), 336 amino acids in USA300 (SAUSA300_0951), and 357 amino acids in MRSA252 (SAR_1022) and other CC30 strains, due to variation in a C-terminal disordered segment comprised of tripeptide repeats. Different isomers produced by the same strain as shown on Western blot (5E), and explained in the text, are attributed to varying degrees of processing of the N-terminal propeptide of the SspA precursor, proSspA.

To evaluate the response of other *S. aureus* strains to LA, we first tested another strain of CA-MRSA known as USA400, and a closely related strain of community acquired MSSA know as MSSA476, which are genetically distinct from USA300. These and other strains used for this analysis are defined in Table 1. After growth in TSB containing 25 µM LA, the culture supernatants of USA300, USA400 and MSSA476 each exhibited accumulation of a protein corresponding to SspA (Fig. 5A) as confirmed by Western blot (Fig. 5B), together with a marked decrease in proGeh, concomitant with appearance of mature Geh (Fig. 5A). Production of SspA by USA300 and MSSA476 appeared to exceed that of USA400, and on several repetitions, USA300 always exhibited more robust production of SspA in response to LA, compared to USA400. Consequently, there are strain dependent differences in induction of the SPC in response to uFFA. We therefore tested additional strains to assess variation in SspA production in response to uFFA (Fig. 5C and D).

Once again, induction of SspA in USA400 was less robust compared to USA300, although the overall impact on the profile of secreted proteins was similar, as noted from the marked reduction in accumulation of proGeh concomitant with induction of SspA (Fig. 5C). Strain WBG10049, representing the Southwest Pacific Clone of CA-MRSA, also exhibited robust induction of SspA (Fig. 5C and 5D) concomitant with disappearance of proGeh, but this characteristic response was not readily observed in MSSA strains Newman and UAMS-1, or in HA-MRSA strain MRSA 252, which have been used as model pathogens to address virulence and gene expression in *S. aureus*. Although these latter strains did not respond to LA, expression of secreted virulence factors in Newman is affected by a unique polymorphism in the *saeRS* regulatory locus [49], while UAMS-1 and MRSA 252 belong to a distinct clade within clonal complex CC30 characterized by altered signaling of the accessory gene regulator *agr* [50], [51], [52]. We therefore tested an additional panel of *S. aureus* isolates obtained from the skin of adult or pediatric atopic dermatitis patients, and strain L528, which is genetically related to UAMS-1, and derived from a patient with infective endocarditis (Table 1).

To varying degrees, with the exception of Newman, which was included as a non-responsive control, each strain exhibited enhanced production of SspA when cultured in TSB supplemented with 25 µM LA. In some of these, including SRI-109, PED1-75, PED2-1, and PED1-53, there was some production of SspA in TSB alone, which was slightly larger than the more abundant isoforms that were produced when cultured with 25 µM LA. This likely reflects our finding that SspA is secreted as an inactive precursor proSspA, and then undergoes a stepwise maturation that requires processing of the N-terminal propeptide by Aureolysin. One of these strains, PED2-1, is a CA-MRSA that corresponds to clonal complex CC97, while the others are MSSA (Table 1). In consideration of these data, we conclude that variable induction of the Staphylococcal Proteolytic Cascade pathway in response to uFFA is a characteristic trait of *S. aureus* clinical isolates, and that this response is particularly robust in the USA300 strain of CA-MRSA, which is known for causing aggressive skin and soft tissue infections.

## Discussion

Our previous work defined the Staphylococcal proteolytic cascade, comprised of a metalloprotease Aureolysin, which undergoes rapid autocatalytic activation, and is then needed to activate the SspA serine protease, which in turn is required to activate the SspB cysteine protease that is co-expressed with SspA in the *sspABC* operon [34], [35], [36], [37], [38]. Cumulatively, the activities of these proteases are consistent with multiple functions related to modulation of adhesion, colonization, tissue invasion and immune evasion, which include degradation of complement and antimicrobial peptides [53], [54], processing of phenol soluble modulins [55], degradation of microbial adhesion proteins [48], [56] and their tissue ligands [34], [57], and processing of kininogen to promote enhanced vascular permeability [34], [58]. The major findings of our present study are that (i), the SPC is induced by antimicrobial unsaturated long chain fatty acids uFFA, in four different genetic backgrounds of CA-MRSA; (ii), amongst CA-MRSA there is variable induction of the SPC in response to uFFA, with USA300 consistently exhibiting a more robust induction of SspA relative to USA400; and (iii) this response was also manifested to varying degrees by clinical MSSA, including pediatric osteomyelitis (MSSA476), infective endocarditis (L528), and several strains recovered from the skin of adult and pediatric atopic dermatitis patients.

With respect to *S. aureus* persistence on skin, Sapienic acid (C16∶1Δ6), which is an isomer of palmitoleic acid (C16∶1Δ9), is the major fatty acid component of human sebum, and separate studies reported minimum inhibitory concentrations of 10–20 µg/ml, and 30 µg/ml respectively, for *S. aureus* [27], [46]. Another study reported the median concentration of palmitoleic acid in human nasal secretions as 12 µg/ml [30], which corresponds to 48 µM. Based on these considerations and our present data, it is apparent that physiologic concentrations of palmitoleic, sapienic and linoleic acid are sufficient to induce the SPC in *S. aureus*, and this is unique to unsaturated fatty acids. Although *S. aureus* does not have the capacity for β-oxidation of fatty acids, exogenous unsaturated fatty acids are transported across the cytoplasmic membrane through as yet unknown mechanisms, and then either directly incorporated into membrane phospholipid, or alternately, can be extended by the Type II fatty acid synthase machinery, prior to incorporation into phospholipid [45], [59]. Unsaturated fatty acids have reduced packing density in membranes, leading to increased membrane fluidity, and our data may reflect a mechanism for sensing changes in membrane fluidity.

It is unclear whether induction of the SPC by unsaturated fatty acids represents an innate immune function of the epithelial barrier to infection, or whether this promotes colonization and virulence. Several studies have implicated a role for secreted proteases of *S. aureus* in promoting biofilm dispersal [60], [61], [62], and it has been suggested that the biofilm mode of growth promotes nasal carriage of *S. aureus* [63]. Specifically, an extracellular serine protease Esp of *S. epidermidis*, which is orthologous to SspA, promoted *S. aureus* biofilm dispersal *in vitro*, and eradicated *S. aureus* nasal carriage in human subjects when administered intranasally. Although it has been debated whether this was due to biofilm dispersal, or degradation of adhesion proteins and their epithelial ligands [64], it supports the contention that nasal carriage could be regulated through induction of the SPC by unsaturated fatty acid in nasal secretions. One study with a limited number of subjects, revealed a wide variance in the level of palmitoleic/sapienic acid in nasal secretions, ranging from 1.8 to 27 µg/ml [30], which corresponds to 7.1 µM to 106 µM. Therefore, it is reasonable to speculate that individuals with higher sapienic acid content would not carry *S. aureus*, due to a combination of antimicrobial activity and induction of the SPC, which would promote biofilm dispersal and interfere with microbial adhesion.

Alternatively, induction of the SPC by uFFA could facilitate the initiation and maintenance of a stable carriage relationship. Importantly, the phenol soluble module (PSM) family of peptides produced by *S. aureus* exhibit antimicrobial activity towards *Streptococcus pyogenes*, which may comprise a mechanism of interference with competing colonizing pathogens, and the PSMα peptides appear to require proteolytic processing by Aureolysin to activate their antimicrobial properties [55], [65]. Moreover, individuals who are colonized by *S. aureus* also have elevated levels of host-derived antimicrobial α- and β-defensins in their nasal secretions [6]. The antimicrobial peptide dermcidin, which is secreted from eccrine sweat glands, can induce expression of the SepA metalloprotease of *S. epidermidis*, and antimicrobial peptides also trigger protease production in *S. aureus* [66]. In this situation, signaling is mediated through the antimicrobial peptide sensor *aps* [67], [68], which also responds to glycopeptides [69], [70], and triggers a global response that leads to modification of membrane lipids and cell wall teichoic acids, which cumulatively promote resistance to antimicrobial peptides. Therefore, induction of the SPC by uFFA could promote colonization, through degradation of host-derived antimicrobial peptides, concomitant with activation of the antimicrobial properties of PSMα1 and PSMα2, which function to eliminate competing pathogens.

In this context, the capacity for uFFA to induce production of secreted proteases appears to exceed that of other environmental signals, and to our knowledge this represents the only environmental stimulus identified thus far, that can lead to accumulation of SspA serine protease as one of the major secreted proteins. Transcriptional profiling studies have evaluated the transcriptome of USA400 CA-MRSA in response to neutrophil microbicides, including azurophile granule proteins, HOCl, and hydrogen peroxide, and although transcription of certain toxin genes, most notably encoding γ-hemolysin, was strongly induced by these signals, there was no major influence on transcription of *sspA* or *aur* [33], and similar observations were noted in assessing the transcriptome of USA300 in response to growth in blood [32]. Therefore, our data allude to a novel signaling pathway that may selectively induce the SPC, which has an important role in modulating adhesion, colonization and invasion [34], [38], [48], [53], [56], [57], [71].

Induction of the SPC was particularly robust in the USA300 strain of CA-MRSA, and in WBG10049 representing the Southwest Pacific clone of CA-MRSA, both of which cause aggressive skin and soft tissue infections, whereas induction of SspA was evident, but consistently less robust in USA400. Intriguingly, USA400 appears to be associated with septicemia, and disruption of the *saeRS* regulator in USA400 strongly attenuated virulence in a bacteremia model, but had no significant influence on virulence in a subcutaneous abscess model [72]. These observations support the contention that the expression and role of subsets of virulence factors is strongly influenced by the site of infection. In this respect, it is noteworthy that *S. aureus* Newman has been used to evaluate the genetic requirements for kidney abscess development [73], [74], establishing the importance of Coagulase and vonWillebrand Factor binding protein in promoting the formation of a fibrin pseudocapsule that impedes migration of neutrophils into the microbe dense core of the abscess [73]. However we, and others, have defined a role for secreted proteases in degrading fibrin, and cell surface fibrinogen binding proteins [34], [48], [57], and abscess tissue has high levels of uFFA, of which the major bactericidal activity is attributed to linoleic acid [31]. Therefore, we must consider that the ability of strain Newman to form large, well defined abscesses could be due in part to its failure to induce protease expression in response to uFFA, as we have demonstrated in this study.

MRSA 252, which is a successful epidemic strain of HA-MRSA [75], also failed to induce the SPC in response to linoleic acid. Strikingly, MRSA 252 was used to evaluate changes in the transcriptome in response to linoleic acid [44], but our data show that it is not representative of *S. aureus*, in terms of its response to uFFA. MRSA 252 belongs to a distinct clade within clonal complex CC30, characterized by accumulation of pseudo genes, and single nucleotide polymorphisms that attenuate virulence, and/or promote enhanced intrinsic resistance to antimicrobial agents [50], [52], [76]. Conversely, WBG10049 is a hypervirulent CA-MRSA that belongs to a separate CC30 clade, and as with USA300, it exhibited robust induction of the SPC in response to linoleic acid.

In summary, we conclude that induction of the SPC in response to uFFA is another factor that should now be considered in addressing the aggressive nature of skin and soft tissue infection caused by some strains of CA-MRSA, and as a general virulence mechanism for most strains of *S. aureus*. Future work will focus on the mechanistic details of induction, including evaluation of the USA300 transcriptome in response to linoleic acid, evaluation of protease null mutants in bacteremia and subcutaneous wound infection models, and the significance of lipase processing by proteolytic activity.
